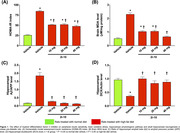# Myeloid Differentiation Factor 2 Inhibition Attenuates Brain Pathologies in the Obese Pre‐diabetic Condition

**DOI:** 10.1002/alz.084629

**Published:** 2025-01-03

**Authors:** Thura Tun Oo, Wichwara Nawara, Busarin Arunsak, Titikorn Chunchai, Nattayaporn Apaijai, Wasana Pratchayasakul, Guang Liang, Nipon Chattipakorn, Siriporn C Chattipakorn

**Affiliations:** ^1^ Center of Excellence in Cardiac Electrophysiology, Chiang Mai University, Chiang Mai Thailand; ^2^ Neurophysiology Unit, Cardiac Electrophysiology Research and Training Center, Faculty of Medicine, Chiang Mai University, Chiang Mai Thailand; ^3^ Cardiac Electrophysiology Unit, Department of Physiology, Faculty of Medicine, Chiang Mai University, Chiang Mai Thailand; ^4^ Chemical Biology Research Center, School of Pharmaceutical Sciences, Wenzhou Medical University, Wenzhou, 325035, China, Zhejiang China; ^5^ Chiang Mai University/Faculty of Medicine/ Department of Physiology, Chiang Mai Thailand; ^6^ Chiang Mai University/Department of Oral Biology and Diagnostic Sciences/Faculty of Dentistry, Chiang Mai Thailand; ^7^ Center of Excellence in Cardiac Electrophysiology Research, Chiang Mai University, Chiang Mai Thailand

## Abstract

**Background:**

Excessive high‐fat diet (HFD) consumption develops the obese pre‐diabetic condition, which initiates neuroinflammation and numerous brain pathologies, resulting in cognitive decline (1). A cinnamamide derivative compound (2i‐10) is recently identified as a novel myeloid differentiation factor 2 (MD‐2) inhibitor, and has been shown to attenuate inflammation via toll‐like receptor 4 (TLR4) signaling pathway (2). However, the effects of 2i‐10 on the neuroinflammation, brain pathologies and cognitive function in the obese pre‐diabetic rats have never been studied.

**Method:**

Male Wistar rats were fed one of two diets for 16 weeks: either a normal diet (ND; n = 8) or a high‐fat diet (HFD; n = 32). At week 13, ND‐fed rats were intraperitoneally administered a vehicle for 4 weeks, while HFD‐fed rats were randomly divided into 4 groups (n = 8/group). Each subgroup was intraperitoneally administrated either vehicle, 10mg/kg/day of 2i‐10, 20mg/kg/day of 2i‐10, or 40mg/kg/day of 2i‐10 for 4 weeks. At the end of week 16, the novel object location (NOL) test was examined in each rat. Brains were rapidly removed to determine hippocampal inflammation, adult hippocampal neurogenesis, brain oxidative stress, dendritic spine density, amyloidogenic pathology.

**Result:**

HFD‐fed rats developed the obese pre‐diabetic condition. Hippocampal inflammation was observed in HFD‐fed rats, as evidenced by increased hippocampal TLR4 and TNF‐α expressions. Moreover, HFD‐fed rats not only had significantly higher hippocampal amyloid‐β/APP ratio and brain MDA levels, but also had significantly lower levels of adult hippocampal neurogenesis, hippocampal dendritic spine density, resulting in cognitive decline. Although all doses of 2i‐10 attenuated systemic insulin resistance, brain pathologies and cognitive impairment, 2i‐10 (40mg/kg/day) showed the highest efficacy in attenuating brain inflammation, brain oxidative stress, amyloid‐β deposition, as well as improving adult hippocampal neurogenesis, hippocampal dendritic spines, resulting in the restoration of cognitive function in obese pre‐diabetic rats (*P* < 0.05, **Figure 1**).

**Conclusion:**

These findings suggest that MD‐2 inhibition could be the potential strategy for attenuating brain dysfunctions in the obese pre‐diabetic condition.